# Regionalization of Hip Fracture Care in Five High‐Income Countries

**DOI:** 10.1111/1475-6773.70002

**Published:** 2025-06-24

**Authors:** Pieter Bakx, Carlos Godoy, Saeed Al‐Azazi, Amitava Banerjee, Nitzan Burrack, David Ehlig, Christina Fu, Laura A. Hatfield, Asa R. Hartman, Nicole Huang, Dennis T. Ko, Lisa M. Lix, Dominik Moser, Victor Novack, Laura Pasea, Feng Qiu, Kieran L. Quinn, Bheeshma Ravi, Therese A. Stukel, Carin A. Uyl‐de Groot, Bruce E. Landon, Peter Cram

**Affiliations:** ^1^ Erasmus School of Health Policy & Management Erasmus University Rotterdam Rotterdam PA the Netherlands; ^2^ Department of Community Health Sciences University of Manitoba Winnipeg Manitoba Canada; ^3^ George & Fay Yee Centre for Healthcare Innovation University of Manitoba Winnipeg Manitoba Canada; ^4^ Institute of Health Informatics University College London London UK; ^5^ Department of Cardiology University College London Hospitals London UK; ^6^ Clinical Research Center Soroka University Medical Center, Faculty of Health Sciences, ben Gurion University of the Negev Be'er Sheva Israel; ^7^ Chair of Health Economics, Policy and Management St. Gallen Switzerland; ^8^ Department of Health Care Policy, Harvard Medical School Boston Massachusetts USA; ^9^ Department of Statistics and Data Science NORC at the University of Chicago Chicago Illinois USA; ^10^ Institute of Hospital and Health Care Administration National Yang Ming Chiao Tung University Taipei Taiwan; ^11^ Schulich Heart Program Sunnybrook Health Sciences Centre, Sunnybrook Research Institute Toronto Toronto Canada; ^12^ ICES Toronto Ontario Canada; ^13^ Department of Medicine University of Toronto Toronto Ontario Canada; ^14^ Temmy Latner Centre for Palliative Care Sinai Health, University of Toronto Toronto Ontario Canada; ^15^ Institute of Health Policy, Management and Evaluation University of Toronto Toronto Ontario Canada; ^16^ Division of Orthopaedic Surgery Toronto Ontario Canada; ^17^ Division of Orthopaedic Surgery Sunnybrook Health Sciences Centre Toronto Ontario Canada; ^18^ Division of General Medicine Beth Israel Deaconess Medical Center Boston Massachusetts USA; ^19^ Department of Medicine, School of Medicine University of Maryland Baltimore Maryland USA

**Keywords:** hip fracture, international comparisons, regionalization

## Abstract

**Objective:**

To describe differences in regionalization of hip fracture care and the volume‐outcome relationship in five countries.

**Study Setting and Design:**

We conducted a population‐based cross‐sectional cohort study in Canada, Israel, the Netherlands, Taiwan, and the United States. Within each country, we stratified patients into quintiles based upon the volume of hip fractures in the hospital where they were treated. We measured regionalization by the proportion of acute‐care hospitals that treated patients with hip fractures and summarized the hospital volume distribution by the ratio of hip fracture volumes for high‐volume hospitals versus low‐volume hospitals. We then examined age‐ and sex‐standardized outcomes and treatment for patients treated at high‐volume and low‐volume hospitals.

**Data Sources and Analytic Sample:**

We used nationally representative administrative data on adults aged ≥ 66 years hospitalized with hip fracture from 2011 to 2019. We followed them until death or 365 days after the discharge date.

**Principal Findings:**

Across countries, the percentage of all acute‐care hospitals that treated hip fractures differed widely (from 37.0% in Canada to 82.8% in Israel), with high‐volume hospitals treating 4–14 times as many hip fractures as low‐volume hospitals. The absolute risk‐adjusted difference in 30‐day mortality for high‐volume compared to low‐volume hospitals ranged between (−1.9% [95% CI, −2.2 to −1.7] in Canada and +1.1% [95% CI, 0.4–1.8] in the Netherlands). The proportion of patients receiving non‐operative fracture treatment was lower in high‐volume hospitals than low‐volume hospitals in all countries (−5.4% [95% CI, −6.5 to −4.3] in Israel to −0.1% [95% CI, −0.5 to 0.3] in the Netherlands).

**Conclusions:**

Hip fracture regionalization differed substantially across countries. The direction and the magnitude of association between greater regionalization and improved patient outcomes were inconsistent across countries.

**Summary**

- What is known on this topic
  ○ Hip fractures are common, costly, and associated with significant morbidity and mortality. Patients typically require prompt evaluation in a hospital followed by surgical repair.
  ○ Many high‐income countries have regionalized high‐complexity procedures such as hip fractures to centers of excellence because of evidence suggesting improved patient outcomes and hospital efficiency at higher‐volume centers.
  ○ There are very few studies comparing the regionalization of discrete procedures across different countries.
- What this study adds
  ○ We found that the degree of hip fracture regionalization differed widely across countries with Canada and the Netherlands having greater regionalization and Israel having lower regionalization.
  ○ We found that country rankings by regionalization rates were sensitive to the way regionalization was quantified.
  ○ High‐volume hospitals had lower mortality in most, but not all, countries.

## 1 Introduction

Hip fractures are a leading cause of morbidity and mortality among older adults [1]. Despite advances in surgical techniques, anesthesia, and postoperative care, the one‐year mortality after hip fracture in older adults is 15%–30% and 40%‐or‐higher in older adults with significant comorbid conditions [2, 3, 4]. Moreover, only a minority of patients manage to regain their pre‐fracture functional status [5], often requiring long‐term assistance with activities of daily living [6].

Many high‐income countries have regionalized high‐complexity medical and surgical procedures to centers of excellence. This is largely because of evidence suggesting improved patient outcomes and hospital efficiency (e.g., shorter hospital length‐of‐stay and lower readmissions) when procedures are performed at higher‐volume centers [7, 8, 9, 10, 11, 12, 13, 14]. However, the United States has generally avoided centralized planning of healthcare delivery in favor of market‐based approaches in which doctors and hospitals are given autonomy regarding which services they provide [15, 16]. Interestingly, we are unaware of any studies that have described regionalization of care for a single disease or procedure across different countries. This is important as prior research has demonstrated that processes of care and patient outcomes after hip fracture differ widely across countries [2, 6]. Hip fractures offer an important “case study” for evaluating how different countries approach regionalization because hip fractures are common, the techniques required to repair hip fractures are relatively standard, and most small‐to‐moderate sized hospitals have the expertise, facilities, and staff required to perform repairs. Furthermore, virtually all individuals with acute hip fractures are hospitalized, thus minimizing selection bias.

In this study from the International Health Systems Research Collaborative (https://projects.iq.harvard.edu/ihsrc), we examine variation in hip fracture regionalization across countries with well‐developed health care systems using population‐representative patient‐level data from the United States, Canada, the Netherlands, Israel and Taiwan. We quantified regionalization using several complementary definitions including hospital hip fracture volume and the ratio of hip fracture volume in high‐volume versus low‐volume hospitals. After evaluating regionalization of hip fracture care within each country, we compared outcomes for patients treated in the highest and lowest hospital volume quintiles within each country to understand cross‐country differences in the volume‐outcome relationship. Our clinical outcomes included hip fracture operative and non‐operative treatments, mortality, hospital length‐of‐stay, and 30‐day readmission rates.

## 2 Methods

### 2.1 Data and Cohort Identification

We identified patients aged 66 years and over who were hospitalized with a primary diagnosis of low‐impact hip fracture between 2011 and 2019 (2014–2019 in the Netherlands) using nationally representative patient‐level data from five diverse high‐income countries; we used a common set of inclusion and exclusion criteria, following a carefully crafted common protocol (Appendix 1). Hip fractures were defined using codes from the International Classification of Disease, Revisions 9 and 10, with local modifications to fit country‐specific practices. Descriptions of each country's data sources and the codes used in this study are included as Appendix 2.

We excluded fractures associated with high‐impact trauma (e.g., traffic accidents), patients with a hospital admission for a hip fracture in the prior 180 days to the index hospitalization, and patients with less than 1 year of pre‐admission data or for whom follow‐up data was unavailable (Appendix 5). Furthermore, we excluded patients with missing demographic data and patients treated in extremely low‐volume hospitals (defined as treating less than six hip fracture patients in a 3‐year period) as such hospitals would not be considered to have true hip fracture management capacity and such patients may represent miscoding (e.g., old hip fractures coded as a new diagnosis). We excluded fee‐for‐service US Medicare beneficiaries without continuous enrollment for the 12 months before and 12 months after index admission. Finally, we excluded US patients enrolled in a Medicare Advantage plan for at least two consecutive months in the 12 months before or after the index admission. Comorbidities were identified using a Manitoba adaptation of the Elixhauser comorbidity index [17]. For this, we used information from the index admission and hospitalizations in the prior 365 days. In Israel, comorbidity measures also included ambulatory care visits due to integrated medical records; in the Netherlands, information on outpatient medication use was added.

*All analyses were used full care episodes*: patients who were transferred from one hospital to another were followed from initial admission to final discharge. For Canada, the Netherlands and Israel patients who were transferred between hospitals were “credited” to the final hospital for sake of volume calculations reflecting practice patterns in these countries. Alternatively, because in Taiwan and the United States we observed patients commonly being transferred after surgery for continued inpatient recovery after surgery (an uncommon practice in other countries), patients in these countries were assigned to the hospital where they received surgery.

### 2.2 Regionalization Measurement

Regionalization has been defined and operationalized in several different ways, most frequently using hospital volume but also using related measures such as the proportion of all patients (or procedures) with a specific condition treated within a certain subset of hospitals [18]. Regionalization may be intentional but may also be the consequence of other factors, including population density, geography and availability of staff and facilities. We measured regionalization in each country using three complementary measures. First, for each country we examined the percentage of all hospitals providing acute, inpatient care that admitted patients with hip fractures, defined as hospitals that admitted six‐or‐more hip fractures during a three‐year period. Second, we evaluated the distribution of hip fracture patients among hospitals providing hip fracture care in each country, as measured by hospital volume. Specifically, we sorted patients hospitalized with hip fractures during three‐year time windows within each country into quintiles based upon the volume of the hospital to which their episode was assigned (Quintile 1‐lowest volume; Quintile 5‐highest volume). Each quintile thus had approximately the same number of patients but a different number of hospitals. To summarize the distribution of hip fracture care in among hospitals providing hip fracture care, we calculated the ratio of hip fractures in the highest‐volume hospitals (Quintile 5) relative to the lowest‐volume hospitals (Quintile 1) in each country for each three‐year period. A high ratio would indicate a larger difference in volume between high‐volume and low‐volume centers within a country. If the number of hospitals providing hip fracture care is constant, a higher ratio would be seen with increased regionalization of care. Third, we measured the number (and percentage) of hospitals treating hip fractures in each country in each calendar year which, in aggregate, captured 90% of the patients hospitalized with hip fractures in that country. Since regionalization practices in each country could change over time and to account for small annual sample sizes in some countries, we aggregated data into 3‐year periods (2011–2013, 2014–2016 and 2017–2019).

### 2.3 Clinical Outcomes

We compared clinical outcomes for patients treated in lower‐volume and higher‐volume hip fracture hospitals in each country. These included risk‐adjusted 30‐day and 1‐year mortality, hospital length of stay and 30‐day readmission post‐discharge among those discharged alive, percentage of patients discharged home, and the percentage of patients with hip fracture repair who received each type of treatment (total hip arthroplasty, hemiarthroplasty, fixation, and non‐operative) across hospital volume quintiles. For patients with multiple procedures during the index admission, we assigned the most extensive repair type (total hip arthroplasty > hemiarthroplasty > fixation), and patients were deemed non‐operative if they lacked procedure codes for all surgical repair types.

### 2.4 Statistical Analysis

First, we compared the demographic characteristics and prevalence of key comorbid conditions for patients treated in high‐volume and low‐volume hospitals within each country; we also examined the proportion of patients in high‐and low‐volume hospitals who were transferred from another hospital for Canada, the Netherlands and the United States, where these data were available.

Second, we compared the concentration of patients hospitalized with hip fractures within each country using the regionalization measures described previously. Third, we compared age, sex, and comorbidity standardized mortality for patients treated in high‐ and low‐volume hospitals by fitting country‐specific logistic regression models with indicators for age (5‐year ranges), sex, and comorbidities. Fourth, we compared hospital length of stay and 30‐day readmission rates, the proportion of patients discharged to home, and the proportion of patients between the quintiles by standardizing the age–sex distribution in quintiles 2–5 to the age–sex distribution in the first quintile in each country. The risk adjustment procedures are explained in further detail in Appendices 3–4. Finally, we compared the age and sex standardized proportion of patients who received each hip fracture treatment strategy (THA, HA, IF, non‐operative management) across quintiles to discern whether hip fracture treatment might differ between higher and lower volume hospitals.

Analysis software included SAS (United States, Canada, Taiwan), Stata (Netherlands) and R (Israel). Analyses were conducted separately in each country. Ethics and data access approvals were obtained in each country following local guidelines.

## 3 Results

The number of hip fracture admissions across the full study period varied across countries from 26,733 in Israel to 2,309,091 in the United States (Table 1 and Appendix 5) The mean age of patients was approximately 82 and approximately 30% were male. Patients treated in high‐volume hospitals (Quintile 5) were older than those in low‐volume hospitals (Quintile 1) in all five countries (by 0.2–0.8 years). The percentage male and prevalence of comorbid conditions were generally similar across quintiles within countries (Table 1). Patients treated in high‐volume hospitals were less likely to originate via an inter‐hospital transfer than patients treated in low‐volume hospitals in Canada and the Netherlands (Table 1); in contrast, patients treated in high‐volume hospitals in the United States were more likely to have been admitted following an inter‐hospital transfer.

**TABLE 1:** Demographic characteristics and prevalence of key comorbid conditions for patients hospitalized with hip fractures 2011–2019 across countries, treated in highest‐volume and lowest‐volume quintile hospitals.

	Canada		Israel		Taiwan		the Netherlands^a^		United States	
	Volume quintile									
	Highest	Lowest	Highest	Lowest	Highest	Lowest	Highest	Lowest	Highest	Lowest
Sample size										
Patients	19,218	18,076	6,567	4,655	30,169	30,052	18,326	16,452	277,878	277,319
Demographics										
Age, years	84	84	83	82	82	82	83	83	84	84
Male, %	28	28	31	33	34	35	27	28	27	28
Comorbidities, %										
Hypertension (uncomplicated)	29	29	68	57	49	39	26	26	62	61
Diabetes (uncomplicated)	8.8	9.1	33	33	27	26	16	15	17	18
Congestive heart failure	4.1	4.4	15	14	6.7	6.6	7.6	7.3	22	21
Hypothyroidism	3.2	3.0	15	11	0.56	0.36	2.4	2.7	26	24
Admissions originating via transfer, %	4.3	5.1	NA^b^	NA^b^	NA^b^	NA^b^	0.50	1.5	13	7.9

^a^ Data for the Netherlands was collected from 2014 to 2019 instead of 2011 to 2019, as was the case with the other four countries.

^b^ Neither Israel nor Taiwan track the origin location of hip fracture admissions.

### 3.1 Regionalization Outcomes

The percentage of hospitals that treated hip fractures among all hospitals providing acute inpatient care differed widely across countries (Appendix 6, Table A1). For example, for the 2017–2019 period, 37.0% of hospitals in Canada treated hip fractures versus 82.8% in Israel (Table A1). The percentage of hospitals treating hip fractures was generally stable within countries over time. 90% of the hip fracture treatment was provided by the 51% (United States) to 71% (Netherlands) highest‐volume hospitals.

**FIGURE 1:**
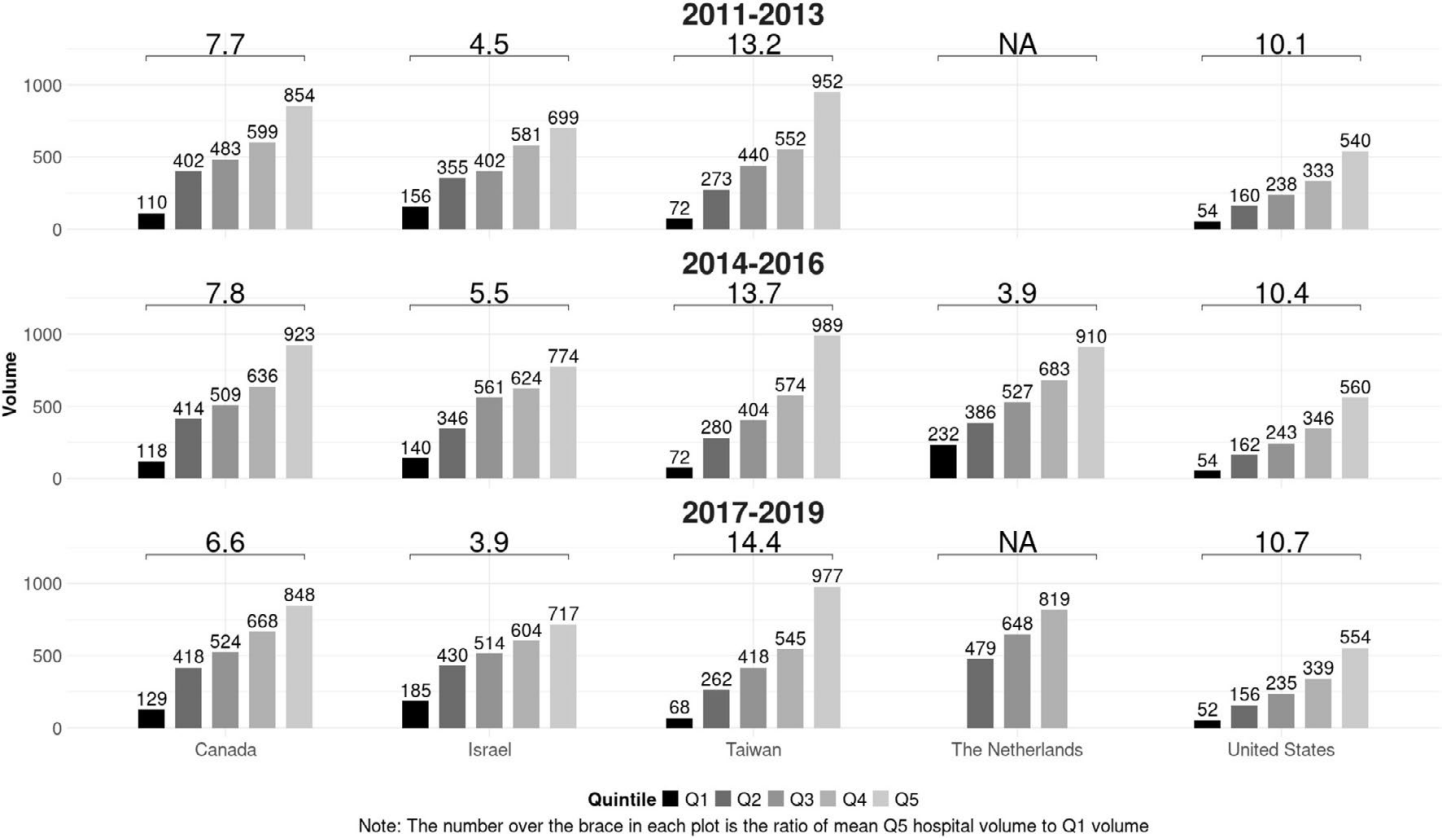
Mean hospital hip fracture volume by quintile across jurisdictions for three‐year bands with ratio of Quintile 5: Quintile 1 volume, 2011–2019. Note to Figure 1: the number over the brace in each plot is the ration of mean Q5 hospital volume to Q1 hospital volume. Data for the Netherlands was collected from 2014 to 2019 instead of 2011–2019, as was the case with the other four countries. Data for the highest‐volume and lowest‐volume quintiles in 2017–2019 could not be reported for the Netherlands because Statistics Netherlands rules do not allow reporting of statistics on fewer than 10 healthcare providers.

For the 2017–2019 period, the ratio of the mean hip fracture volume for the highest‐volume quintile versus the lowest‐volume quintile hospitals ranged from 3.9 in Israel to 14.4 in Taiwan (10.7 in the United States (Figure 1)); this suggests a higher degree of regionalization in Taiwan as compared to Israel. Looking longitudinally, this volume ratio increased slightly between 2011–2013 and 2017–2019 in the United States (10.1–10.7) and Taiwan (13.2–14.4) but decreased in Israel (4.5–3.9) and Canada (7.7–6.6) (Figure 1). Furthermore, quintile‐specific average volumes in 2017–2019 were lowest in the United States (52 cases in 3 years in Quintile 1; 554 in Quintile 5) and the highest in Taiwan (977 in Quintile 5) and Israel (185 in Quintile 1). In Taiwan, the 55% highest‐volume hospitals treated 90% of the total hip fracture volume (Figure A1), which is the lowest percentage in all five countries, suggesting greater regionalization. By contrast, the 70% highest‐volume hospitals treated 90% of the total hip fracture volume in the Netherlands, suggesting less regionalization.

### 3.2 Clinical Outcomes

**FIGURE 2:**
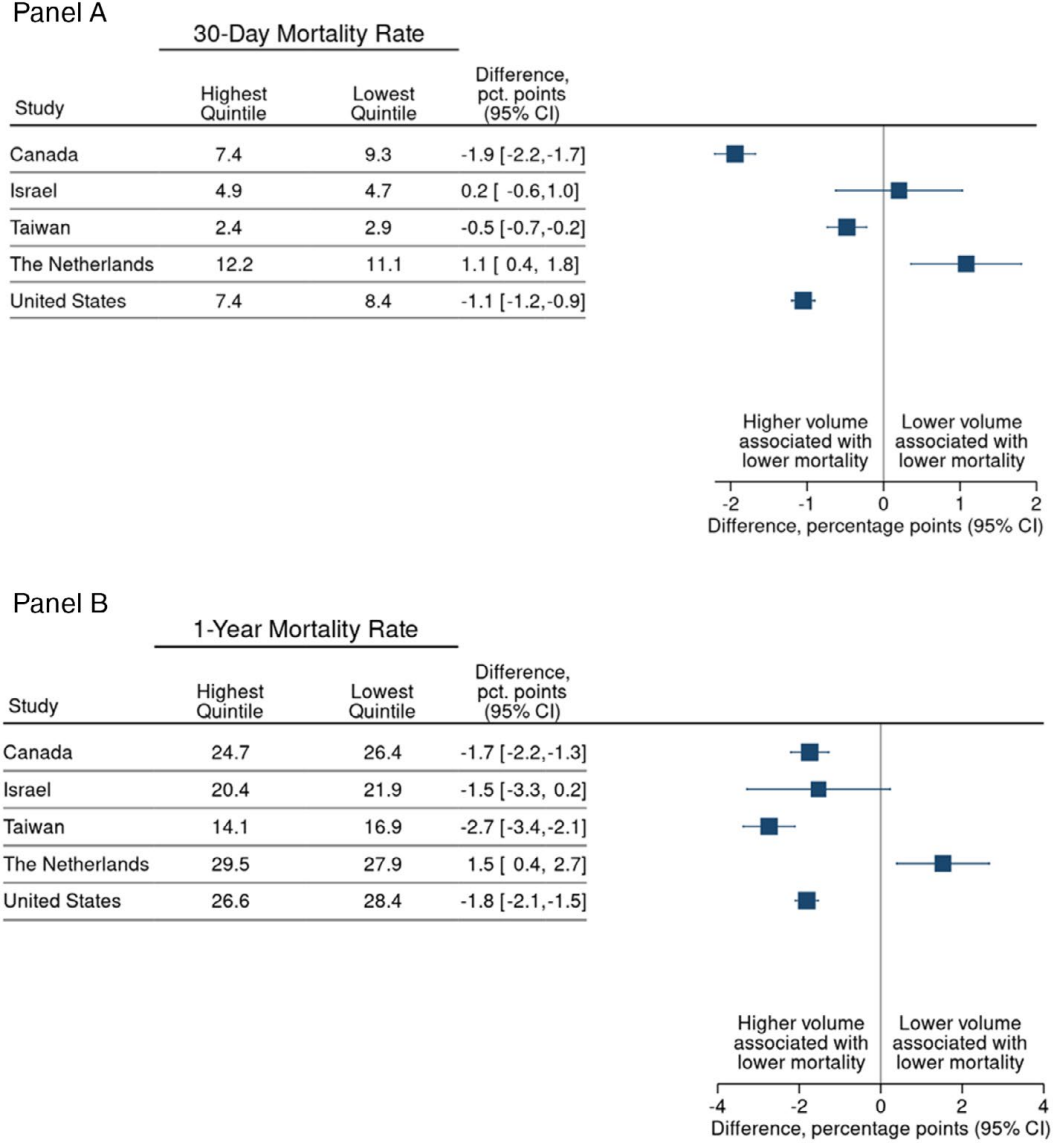
30‐day mortality rate (%) for highest and lowest hospital volume quintiles, adjusted for age, sex and comorbidity. (A) 30‐day mortality, (B) 1‐year mortality.

Risk‐adjusted mortality rates were lower in high‐volume hospitals than in low‐volume hospitals in all countries with one exception (Figure 2A and Appendix 6, Table A2). For example, the 30‐day mortality rate in Canada was 1.9% lower (95% confidence interval (CI): −2.2% to −1.7%) in high‐volume hospitals (7.4%) compared to low‐volume hospitals (9.3%). Similarly, in Taiwan, high‐volume hospitals had lower 30‐day mortality rates than low‐volume hospitals (2.4% vs. 2.9%; difference −0.5% [95% CI: −0.7% to −0.2%]) while rates in the United States were 7.4% versus 8.4%, respectively (difference −1.1% [95% CI: −1.2% to −0.9%]) (Figure 2A). The Netherlands presents a contrasting scenario where high‐volume hospitals have a higher 30‐day mortality rate (12.1%) than low‐volume hospitals (11.1%), even after risk adjustment. 1‐year mortality rates followed a similar pattern to 30‐day mortality in most countries (Figure 2B).

**FIGURE 3:**
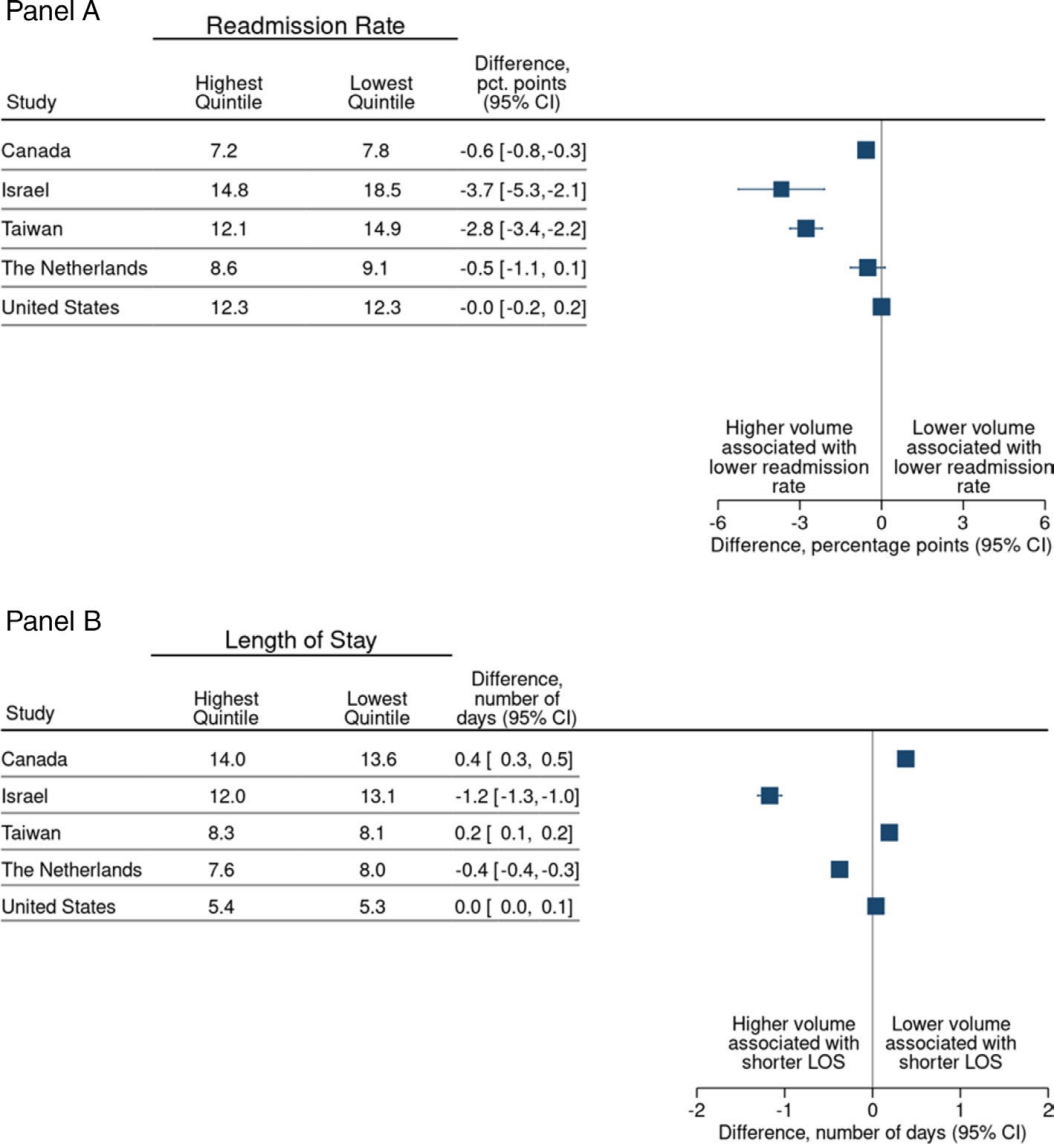
30‐day readmission rate (%) and hospital length of stay for highest and lowest hospital volume quintiles, adjusted for age, sex and comorbidity. (A) Readmission rate, (B) length of stay (LOS).

30‐day hospital readmission rates were lower in high‐volume hospitals than in low‐volume hospitals in most countries (Figure 3A and Appendix 6, Table A2). For example, the 30‐day readmission rate in Israel was 14.8% in high‐volume and 18.5% in low‐volume hospitals (difference 3.7%; 95% CI: −5.3% to −2.1%) and in Taiwan 12.1% and 14.9% (difference −2.8% and 95% CI: −3.4% to −2.2%). There were negligible differences in the Netherlands and the United States (Figure 3A). Looking across countries, there was no consistent relationship between hospital volume and length‐of‐stay (Figure 3B). Patients treated in high‐volume hospitals were more likely to be discharged home relative to those treated in low‐volume hospitals in all four countries for which data were available (Appendix 6, Table A2 and Figure A2).

Looking at the type of hip fracture treatment, hemiarthroplasty and fixation were performed more frequently in high‐volume hospitals than in low‐volume hospitals in most countries, with the Netherlands being a notable exception for fixation (49.5%–52.1%) (Table 2). Patients treated at low‐volume hospitals more often received non‐operative management relative to patients in high‐volume hospitals across all countries. There was no clear pattern of higher or lower use of total hip arthroplasty among high‐volume hospitals across countries (Table 2).

**TABLE 2:** Hip fracture treatment by country for patients treated in highest‐volume and lowest‐volume quintile hospitals in %, 2011–2019.

Treatment received	Country	Highest quintile (%)	Lowest quintile (%)	Difference (%)	Lower bound of difference, 95% CI	Upper bound of difference, 95% CI
Total hip arthroplasty incidence	Canada	7.7	9.1	–1.4	–2.0	–0.78
	Israel	5.2	4.7	0.46	–0.39	1.3
	Taiwan	0.73	0.44	0.29	0.17	0.41
	the Netherlands^a^	4.0	5.9	–1.9	–2.4	–1.4
	United States	4.7	4.3	0.36	0.25	0.47
Hemiarthroplasty incidence	Canada	32	29	3.2	2.1	4.3
	Israel	26	25	0.67	–1.2	2.6
	Taiwan	40	39	0.70	–0.31	1.7
	the Netherlands^a^	36	31	4.9	3.7	6.1
	United States	30	30	0.35	0.06	0.63
Fixation incidence	Canada	56	54	2.3	0.80	3.8
	Israel	64	60	4.3	1.3	7.2
	Taiwan	56	53	3.0	1.9	4.2
	the Netherlands^a^	49	52	–2.6	–4.1	–1.1
	United States	59	58	0.80	0.40	1.2
No intervention occurrence	Canada	4.7	8.7	–4.0	–4.5	–3.4
	Israel	5.2	11	–5.4	–6.5	–4.3
	Taiwan	3.4	7.4	–4.1	–4.5	–3.7
	the Netherlands^a^	3.2	3.3	–0.09	–0.47	0.30
	United States	6.0	7.6	–1.5	–1.6	–1.4

^a^ Data for the Netherlands was collected from 2014 to 2019 instead of 2011 to 2019, as was the case with the other four countries.

## 4 Discussion

In an analysis of 2,797,860 patients using population‐based data from five high‐income countries, we found substantial between‐country differences in the extent to which hip fracture care was regionalized among hospitals. There were large between‐country differences in the percentage of hospitals treating hip fractures, in the distribution of hip fracture volumes within the group of hospitals providing treatment, and differences in the proportion of “hip fracture hospitals” that treated 90% of the total hip fracture volume in each country. In evaluating the potential impact of higher versus lower regionalization as captured by hospital hip fracture volume, we found an inverse association between hospital volume and mortality in four of five countries and evidence that low‐volume hospitals were more likely to provide non‐operative management. Taken together, our results provide a novel perspective on how hip fracture care is organized across countries.

Several findings warrant further interpretation. First, it is important to comment on our efforts to compare regionalization across countries. Foundational studies demonstrating improved surgical and procedural outcomes at higher volume hospitals, leaders in government, hospital accreditation bodies, and the quality community have championed “regionalization of care” (also referred to as “centralization”) to high‐volume hospitals and centers of excellence [7, 8, 9, 10, 11, 12, 13, 14]. Yet, we are unaware of studies that have attempted to compare regionalization for a discrete diagnosis or condition across different countries.

We used three different measures to assess regionalization of hip fractures across countries: (1) the proportion of acute‐care hospitals in each country that treated hip fractures, (2) the ratio of the average volume in high‐volume (Quintile 5) versus low‐volume (Quintile 1) hospitals, and (3) the percentage of hospitals in each country treating hip fractures which, in aggregate, captured 90% of the total hip fracture volume in that country during a given calendar year [19].

Canada and Taiwan had the lowest percentage of their total acute‐care hospitals treating hip fractures, suggesting greater regionalization with hip fracture care concentrated in a smaller group of hospitals, while Israel and the United States had a higher percentage of hospitals treating hip fractures, suggesting less regionalization. Alternatively, the Quintile 5: Quintile 1 volume ratio was highest in Taiwan and the United States, suggesting greater concentration of hip fracture volume within the highest‐volume hospitals in these countries, with lower ratios in Canada and Israel suggesting lesser concentration within the subgroup of acute‐care hospitals providing hip fracture care, which is relatively small in Canada. Taiwan was notable for having both a low proportion of hospitals treating hip fractures (47% in 2017–2019), a high Quintile 5: Quintile 1 volume ratio (14.4 in 2017–2019) and a smaller proportion of hospitals treating 90% of all hip fracture cases (51% in 2017–2019) suggesting high regionalization of hip fracture care by both measures. The three measures showed different relative rates of regionalization for the other countries, which might be because of different geographical characteristics and different policy instruments, e.g., minimum volume requirements or financial incentives that are used to foster regionalization, leading to different hospital volume patterns. Our prior work revealed that Taiwan (high regionalization in the current analysis) had low 30‐day and 1‐year hip fracture mortality, but we also found that Israel (low regionalization in the current analysis) had low mortality, while Canada (high regionalization) had relatively high mortality [2]. Taken in aggregate, the findings do not provide conclusive evidence that hip fracture regionalization is associated with overall cross‐country differences in hip fracture mortality.

It is interesting to consider our regionalization results in the context of what is known about regionalization efforts in each country. In Ontario (Canada), hip fracture care is included in a set of conditions that are covered by the province's quality based procedures (QBPs) program that was introduced in 2012 [20]. While the QBP program was designed to better link hospital payment to both hip fracture quality and hip fracture volume, the program did not specifically attempt to regionalize or centralize hip fracture care [21]. Manitoba initiated a provincial hip fracture regionalization strategy in 2012 that included reducing the number of hospitals repairing hip fractures in the region from four to three; an empirical evaluation found that the implementation reduced surgical delays, but did not significantly reduce mortality, complication rates, or hospital length‐of‐stay [22]. While some US states previously had state‐level statutes *certificate of need* (*CON*) processes that were used to regulate the number of hospitals offering certain complex surgical procedures (e.g., cardiac surgery), CON regulations were repealed by many states in the 1980s and 1990s, and CON regulations never pertained to hip fractures [23, 24]. In the Netherlands, to our knowledge, no strategy for regionalization of hip fracture care has been in place, yet hip fracture care may have been influenced by other efforts, e.g., policies facilitating or incentivizing hospital mergers and closure of smaller facilities.

Hip fracture is an ideal “tracer” condition for several reasons that merit consideration. First, hip fractures are common, meaning that many small‐to‐moderate sized hospitals will routinely admit older adults with hip fractures [19, 25]. Second, the surgical management and anesthesia techniques required to repair hip fractures are relatively standard, meaning that most small‐to‐moderate sized hospitals have the expertise, facilities, and staff required to perform repairs. Third, the optimal time window for repairing a hip fracture is 24–48 h [3], meaning that it is feasible to transport patients, even those in relatively remote areas, to a distant center of excellence if health systems choose such a strategy [26]. Fourth, patients with hip fractures can receive one of four treatment approaches (total hip arthroplasty, hemi‐arthroplasty, fixation, or non‐operative management) which provide additional nuance when evaluating treatment patterns [27]. Finally, virtually all individuals with acute hip fractures are hospitalized, thus minimizing selection bias that can be introduced where admission to the hospital is more discretionary and may differ across countries.

Second, our finding of an inverse relationship between hip fracture volume and mortality in four out of five countries reinforces prior work on the volume‐outcome relationship for hip fracture patients within one country [7, 8]. Our finding of higher mortality in higher volume hospitals in the Netherlands is surprising. Prior work using other data reported no volume‐outcome relationship [28]; one potential explanation is different measures of comorbidity adjustment that we used (outpatient medication use in the Netherlands versus Elixhauser measures based on inpatient comorbidities in other countries). Another explanation is related to the considerably higher average volume of hospitals in Quintile 1 in the Netherlands compared to other countries: these volumes might be sufficiently high to materialize the advantages associated with a high volume. Finally, there were fewer inter‐hospital transfers in the Netherlands.

Third, it is important to note some of the variation in hip fracture treatment across high‐and low‐volume hospitals, particularly the share of patients who received non‐operative management. Non‐operative management is typically reserved for patients with multi‐morbidity, frailty, cognitive impairment, and poor pre‐fracture health status. The percentage of patients receiving non‐operative management was more than twice as high in low‐volume hospitals in Canada, Israel and Taiwan than in hospitals in the highest‐volume quintile in each country; the differences were smaller in the United States and the Netherlands. The consistency of this finding could reflect differential practice patterns in low‐volume hospitals, but more likely reflects that patients with poor prognosis are preferentially treated in hospitals close to their home, which may be smaller, rather than being transported to larger centers.

Fourth, for countries where data on discharge destination is available, we see that high‐volume hospitals are less likely to discharge patients home than low‐volume hospitals. We speculate that this difference in discharge destination may be because high‐volume hospitals may be part of a regional hip fracture “system” and thus able to provide easy access for patients to post‐acute‐care (PAC) facilities whereas lower hospitals may not. In addition, higher‐volume hospitals may have relatively high throughput on their orthopedic services, choosing to send patients for rehabilitation more commonly in some form of post‐acute care.

Our study has several limitations. First, we lacked detailed information on fracture type or location, as well as detailed information on patients' pre‐fracture functional status. While these factors may play a crucial role in explaining individual‐level differences in treatment and outcomes, these factors likely differ to a small extent within countries. Second, hospital volumes were calculated based on included episodes only, and thus we assume that excluded episodes are distributed similarly across hospitals. Third, the hospital grouping into quintiles may also have been affected by the definition of a hospital location, hospital openings, mergers, and closures during any of the three‐year blocks for which quintiles were calculated. These events add noise to the data and may lead to attenuation bias and thus underestimation of the true relationship between hospital volume and outcomes. Hence, the associations we estimated are likely to be conservative. Furthermore, the US data do not contain Medicare Advantage patients, and the data from Israel were limited to patients enrolled in the largest health insurance program (Clalit), which represents approximately 50% of the population. While we believe that this did not impact our findings, our results should be interpreted with this limitation in mind. Finally, data for 2011 and 2012 were missing for the Netherlands, nor did we observe staffing levels and patient preferences for treatment in any of the counties.

## 5 Conclusion

Using an array of measures, we found substantial between‐country differences in the regionalization of hip fracture care that have not been reported previously. To the extent that care is more efficient and patient outcomes are improved with regionalization, our results suggest that care could be improved in several countries if regionalization efforts were enhanced.

## Conflicts of Interest

The authors declare no conflicts of interest.

## Supporting information


Data S1.

## Data Availability

The data that support the findings of this study are available from the data owners in each of the countries. Restrictions apply to the availability of these data, which were used under license for this study. Data are available from the author(s) with the permission of the data owners in each of the countries.
